# DNA methylation biomarkers for the diagnosis and treatment management of breast cancer: where are we now?

**DOI:** 10.1080/17501911.2025.2548755

**Published:** 2025-08-21

**Authors:** Sarah Williams, Susan J. Clark, Ruth Pidsley, Clare Stirzaker

**Affiliations:** a Epigenetics Research Laboratory, Cancer Ecosystems Program, Garvan Institute of Medical Research, Sydney, NSW, Australia; b School of Clinical Medicine, UNSW Medicine and Health, Sydney, NSW, Australia

**Keywords:** DNA methylation, breast cancer, biomarker, epigenetics, prognosis, treatment, detection, circulating tumor DNA

## Abstract

Breast cancer is one of the most commonly diagnosed cancers worldwide and is a significant contributor to the global cancer burden. It is a clinically heterogeneous disease and reliable tools are needed to support treatment decisions, including patient risk, prediction of therapeutic response and monitoring patients throughout their cancer journey. DNA methylation alterations are an early occurring, highly pervasive and stable modification during tumorigenesis, making DNA methylation an attractive target for the development of biomarkers. In this review, we first provide an overview of DNA methylation and explore its role in cancer, with an emphasis on breast cancer. We then focus on the potential use of tissue- and blood-based DNA methylation biomarkers to inform clinical decision-making in breast cancer paradigms: diagnosis; disease sub-typing; prediction of therapy response to neoadjuvant chemotherapy, endocrine therapy and immunotherapy; prognosis; and the tumor microenvironment. We highlight the significant progress achieved over recent decades in the development of DNA methylation-based biomarkers for breast cancer care. We end by discussing how the integration of advanced research methodologies and bioinformatic tools, and their incorporation into liquid biopsy platforms and ctDNA assays, offer promising opportunities for these biomarkers to be widely adopted in clinical practice.

## Plain Language Summary

Breast cancer is one of the most common cancers worldwide. There are many risk factors, tumor types and responses to treatment vary greatly between patients. This means that clinicians need effective tests to help with treatment decisions and monitor patient progress over time. DNA methylation is a chemical ‘tag’ on the DNA that occurs early in tumor development and remains stable as tumors grow. This review explains what DNA methylation is and how it goes wrong in breast cancer. We show how DNA methylation can be used to help diagnosis, predict response to treatment, and for long-term monitoring of all breast cancer patients, using tissue and blood samples.

## 1. Introduction

Epigenetics is the study of modifications to DNA that regulate the genome without altering the DNA sequence. The primary epigenetic mechanisms that work in concert to regulate gene expression include DNA methylation, histone post-translational modifications, chromatin organization and the higher-order three-dimensional structure [1,2]. It is well established that epigenetic mechanisms play a crucial role in the differentiation of cells and tissues in multicellular organisms [3]. In differentiated cells, epigenetic mechanisms facilitate the regulation of gene expression patterns required for the function of each cell type [4]. Aberrations in the epigenetic regulation of gene expression occur in a variety of diseases [5–7]. Of note, ‘non-mutational epigenetic reprogramming’ has recently been promoted as a key hallmark of cancer, implicated in the progression of a malignant phenotype across all tumor types [8–10].

The most extensively studied epigenetic modification is DNA methylation [11]. The advent of genome-wide DNA methylation sequencing technologies has enabled the study of cancer-specific DNA methylation changes [12], providing greater insight into underlying mechanisms, location of cancer-specific changes and inter-individual differences. Indeed, whole-genome methylation profiling has revealed methylation signatures of therapy response and resistance, leading to novel therapeutic strategies, as well as providing biomarkers for early detection and diagnosis, prognosis and monitoring.

In this review, we focus on DNA methylation in breast cancer, one of the most commonly diagnosed malignancies among women and a leading cause of cancer-related mortality [13]. We provide an overview of DNA methylation and its role in cancer before focusing on the development of DNA methylation biomarkers to inform clinical decision-making in breast cancer care paradigms.

## 2. DNA methylation

### 2.1. Biology of DNA methylation

**Figure 1.**
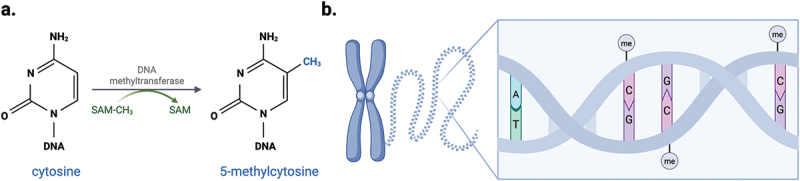
DNA methylation occurs at cytosine-guanine dinucleotides.

DNA methylation is the addition of a methyl group (CH_3_) at the carbon-5 position of a cytosine base, resulting in the formation of 5-methylcytosine (5mC) (Figure 1(a)). The majority of DNA methylation occurs in the context of cytosine-guanine dinucleotides, termed CpG sites [14] (Figure 1(b)). It is well-established that DNA methylation plays a key role in core biological processes, including normal cell development [15,16], X chromosome inactivation [17,18], regulation of tissue-specific gene expression and imprinted alleles [19–21].

(a) DNA methyltransferase enzymes catalyze the addition of a methyl group to the fifth carbon position of cytosines to form 5-methylcytosine (5mC). (b) DNA methylation (me) typically occurs in the context of cytosine-guanine (CpG) dinucleotides. SAM = S-adenosyl methionine. Figure created in BioRender. Williams, S. (2025) https://BioRender.com/smngozv

The human genome encodes five DNA methyltransferases (DNMTs): DNMT1, DNMT2, DNMT3A, DNMT3B and DNMT3L, which catalyze DNA methylation via the transfer of a methyl group from S-adenosyl methionine (SAM) (Figure 1(a)) [14]. DNMT1, also termed the *maintenance* DNMT, plays a principle role in preserving the pattern of parental methylation on newly synthesized daughter DNA strands during DNA replication [22]. In addition, DNMT1 is recruited to sites of DNA damage, suggestive of a role in the repair of DNA methylation [23]. DNMT3a and DNMT3b participate in *de novo* methylation, which occurs independently of DNA replication, and is vital for normal development and cell-type specific differentiation [24]. Currently, the mechanism that determines which DNA sequence the DNMT3 isoforms methylate is unclear [14]; however, evidence suggests CpG sites are preferentially methylated and binding is influenced by the surrounding sequence [25,26]. Another member of the DNMT family, DNMT2, originally thought to be a DNA methylating enzyme, catalyses the methylation of tRNA [27].

DNA can also lose methylation, and this process can broadly be characterized as either passive or active DNA demethylation [28]. Passive demethylation occurs in dividing cells, when the inhibition or dysfunction of DNMT1 causes newly incorporated cytosines to remain unmethylated [14]. In contrast, active demethylation requires enzymatic reactions and can occur in both dividing and non-dividing cells. Two major mechanisms for active demethylation have been described. First, ten-eleven translocation enzymes mediate demethylation via the addition of a hydroxyl group onto the methyl group of 5mC to form 5-hydroxymethylcytosine (5hmC) [29]. Iterative oxidation then converts 5hmC to cytosine [30]. In the second proposed mechanism, deamination of the amine to a carbonyl group effectively converts 5mC to thymine via AID/APOBEC (activation-induced cytidine deaminase/apolipo-protein B mRNA editing enzyme complex). In turn, the base excision repair pathway is invoked to restore the base to cytosine [31].

In mammals, DNA methylation typically occurs in the context of cytosine-guanine (CpG) dinucleotides [14]. The human genome contains approximately 28 million CpG sites, approximately 70% of which are methylated in normal somatic cells [32]. CpG sites are not evenly distributed; CpG pairing occurs at a lower frequency than expected across the bulk of the genome interspersed with regions of more abundant CpG sites termed ‘CpG islands,’ which are typically 500–1000 base pairs in length and commonly span promoters of genes [33]. Promoter CpG-islands are typically unmethylated in normal cells and exist in a transcriptionally permissive state that allows the binding of transcription factors and RNA polymerase II [34,35]. In contrast, gene bodies can be extensively methylated [36]. This intragenic DNA methylation has been demonstrated to preclude spurious transcription initiation by preventing ad hoc RNA polymerase II binding [37]. Distal regulatory elements, such as enhancers, also play a key role in ensuring tissue-specific gene expression by serving as anchor points for transcriptional elements [38]. Typically, unmethylated enhancers allow binding of trans-activating factors, in turn promoting transcription of target genes [39].

### 2.2. DNA methylation alterations in cancer

**Figure 2.**
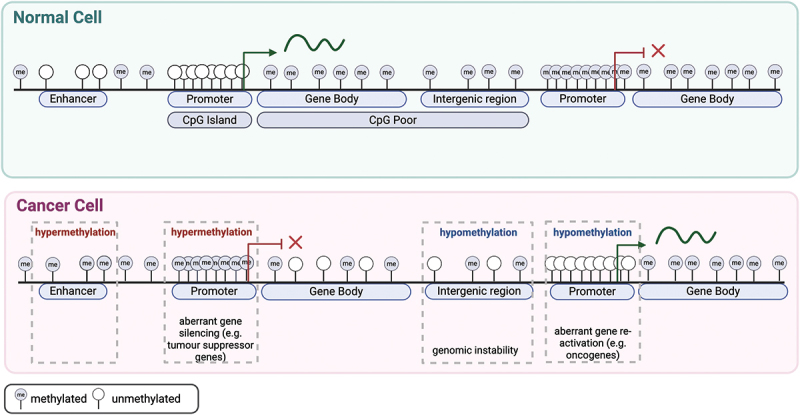
Normal and cancer cells have distinct DNA methylation profiles.

The advent of bisulfite-based sequencing to map DNA methylation at single molecule levels in the early 1990s [40], followed by genome-wide bisulfite-based technologies [12], enabled investigation of the DNA methylome of cancer cells. It is now well-established that DNA methylation patterns in normal cells can be significantly altered in the initiation and progression of cancer [10]. Two types of DNA methylation change contribute to the oncogenic phenotype; namely, genome-wide hypomethylation of repetitive elements, CpG-poor intergenic regions and promoters, including oncogenes, and hypermethylation of CpG-island gene promoter regions [10] (Figure 2).

A schematic representation of the DNA methylome and the changes that occur in cancer cells. The majority of CpG islands occur at gene promoters. In normal cells (green), intergenic, CpG-poor regions are typically methylated, whereas CpG poor enhancers are often unmethylated. Contrastingly, in cancer cells (red) DNA hypermethylation often occurs at CpG islands, resulting in aberrant gene silencing of tumor suppressor genes. Concomitant hypomethylation contributes to the aberrant expression of oncogenes and genomic instability. Figure adapted from [12] and created in BioRender. Williams, S. (2025) https://BioRender.com/860zksx

DNA hypomethylation was the earliest characterized change in DNA methylation in cancer cells [41,42]. In normal cells, CpGs in the genome that are not located in CpG-rich regions, are approximately 60–80% methylated; however, in cancer this methylation declines to 40–60% across the genome [43]. This global hypomethylation in cancer cells contributes to loss of imprinting, chromosome instability, aberrant expression of proto-oncogenes and increased mutagenesis [44,45]. Multiple studies investigating the depletion of DNMT1 observe increased aneuploidies and tumor induction, providing further support for the role of hypomethylation in chromosomal fragility [46,47]. Moreover, the expression of DNMTs has been found to be disrupted in various tumor types, providing a potential mechanism for the observed aberrant methylation [48,49].

In parallel, promoter CpG islands become hypermethylated during tumorigenesis, silencing the expression of tumor suppressor genes [32], first reported for the retinoblastoma tumor suppressor gene [50]. Polycomb marked CpG island promoters are also commonly hypermethylated in cancer cells [51] and the pattern of methylation at these regions is cancer-type specific [52]. In a landmark paper, Esteller and colleagues conducted methylation analyses on 15 tumor types and demonstrated that each cancer displayed a unique profile of promoter hypermethylation [53]. Extensive research in the last two decades has confirmed that DNA methylation changes are tumor-type specific [54,55]. Interestingly, hypermethylation of promoter CpG islands is not isolated to single loci in cancer; indeed, multiple contiguous regions can become co-ordinately silenced and aberrantly methylated [56,57].

## 3. Breast cancer

### 3.1. Breast cancer overview

Breast cancer is one of the most commonly diagnosed cancers worldwide and is the leading cause of cancer mortality in women [58]. The global incidence of breast cancer has been rising with an estimated annual increase of 0.33% per year and this trend is likely to continue given population growth and an aging population [59]. There are many factors that increase the risk of developing the disease, including genetic predispositions [60] and lifestyle, notably, late age pregnancy, nulliparity and obesity [61–63]. Whilst breast cancer is curable in 70–80% of patients with early-stage disease (defined as cancer contained in the breast or that has only spread to axillary lymph nodes), metastatic breast cancer is considered incurable [64].

Currently, breast cancer is diagnosed through clinical examination alongside imaging (mammography and/or ultrasound) and pathological assessment [65]. Pathological characteristics of the disease, such as histological type and grade and hormone receptor status, stratify tumors into distinct biological and molecular subgroups, which inform patient treatment and management [66]. In 2000, Perou and Sorlie [67] investigated gene expression patterns in breast tumors, distinguishing four subtypes of breast cancer: Luminal A, Luminal B, basal-like and HER2-enriched. With the advent of microarray expression profiling studies, this subtyping has extended to five primary subgroups with the inclusion of a claudin-low subgroup [68]. More broadly, breast tumors can be classified by hormone receptor status, based on the presence or absence of estrogen receptor (ER), progesterone receptor (PR) and human epidermal growth factor receptor 2 (HER2) enrichment (summarized in Table 1). Tumors expressing ER, PR and/or HER2 are considered hormone receptor-positive breast cancers, whereas tumors that do not express ER, PR or HER2 are termed triple-negative breast cancer (TNBC) [64].

**Table 1.** Molecular subtypes of breast cancer and their hormone receptor status.

Intrinsic Subtype	ER		PR	HER2 enrichment	Frequency	Prognosis
Luminal A	+	and/or	+	–	60–70%	Good
Luminal B	+	and/or	+	+	10–20%	Good – Intermediate
HER2-enriched	–	and	–	+	5–15%	Intermediate – Poor
Basal-like/TNBC*	–	and	–	–	15–20%	Poor

* the majority of basal-like tumors are triple negative (~80%), however the terms are not strictly interchangeable.

Frequency of cases from [69].

ER: Estrogen Receptor, PR: Progesterone Receptor, HER2: Human Epidermal Growth Factor Receptor 2, TNBC: Triple Negative Breast Cancer.

### 3.2. DNA methylation in breast cancer

Some of the first studies addressing epigenetic modifications in breast cancer investigated promoter hypermethylation in key candidate breast cancer genes, including the breast cancer gene *BRCA1* [70] and *Estrogen Receptor 1* (*ESR1*) [71]. Since this time, global DNA methylation changes in breast cancer have been documented, with observations of global hypomethylation [72] alongside regional hypermethylation [73,74]. Multiple studies have identified that this epigenetic deregulation is present at the very early stages of breast cancer progression and is associated with transcriptional aberrations [75–77]. As summarized by Lo and Sukumar [78] many promoter regions have been identified as hypermethylated in breast cancer, encompassing various biological functions, including cell cycle regulation, apoptosis, DNA repair, hormone regulation and more.

## 4. DNA methylation biomarkers in breast cancer

**Figure 3.**
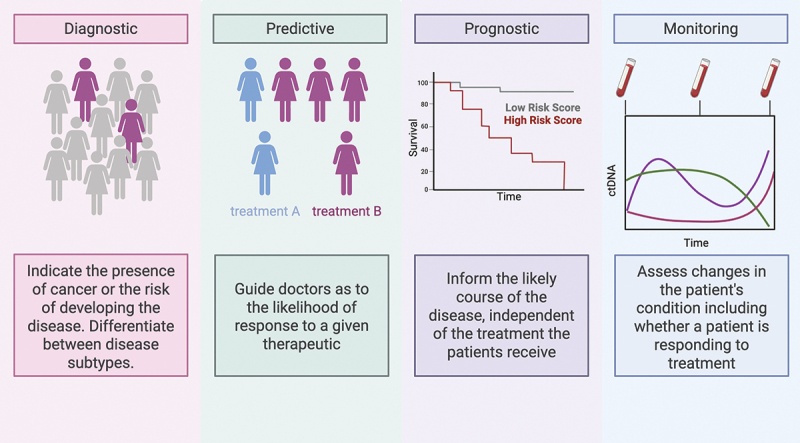
Examples of the potential application of DNA methylation biomarkers for breast cancer management.

The term ‘biomarker’ denotes a measurable indicator of a biological state, which, in the context of breast cancer, includes markers associated with cancer biology [79]. One example of a cancer DNA biomarker is a germline or somatic genetic mutation. However, there are currently a limited number of mutations defined in breast cancer [80]. Indeed, up to 43% of patients do not have germline or somatic mutations at known breast cancer risk genes [81]. In contrast, DNA methylation is an early occurring, highly pervasive and stable modification in breast cancer [78]. These properties make breast cancer-associated DNA methylation changes an attractive area for the development of biomarkers [82]. Across the breast cancer care paradigm, biomarkers are needed for diagnosis, disease sub-typing, prediction of therapy response, prognosis and long-term monitoring (Figure 3).

Biomarkers, including DNA-methylation-based biomarkers, can address various aspects of breast cancer management. At diagnosis, tissue-based biomarkers help to define the subtype of the disease as well as assess individuals at risk of developing the disease. In the breast cancer setting, these tissue-based biomarkers can help predict whether a patient will respond to a given treatment (e.g., chemotherapy, endocrine therapy and/or immunotherapy) and indicate the prognosis of the disease. Using blood-based biomarkers such as circulating tumor DNA (ctDNA), serial assessment can identify changes in the patient’s condition across time. Figure created in BioRender. Williams, S. (2025) https://BioRender.com/n63srcv

### 4.1. Diagnostic biomarkers

Population screening via mammography, aimed at detecting the disease at an early stage has been instrumental in improving outcomes for breast cancer patients [83]. However, the effectiveness of mammography is age-dependent [84] and it has also been reported that racial background affects the rate of false-positives [85]. Immunohistochemical (IHC) testing of estrogen and progesterone receptors, is another current diagnostic method with shortfalls; a systematic review conducted by Hammond and colleagues [86] reported that up to 20% of IHC staining results may be inaccurate. Therefore, there is a clinical need to develop novel, improved approaches to breast cancer screening and subtyping.

#### 4.1.1. Biomarkers for breast cancer diagnosis/detection

Early detection and diagnosis are pivotal for managing breast cancer [64]. Many studies investigating DNA-methylation-based diagnostic tests in breast cancer have used blood-based samples [87,88], explored in later sections in this review. Utilizing tissue-based samples, Downs and colleagues [89] developed a breast cancer-specific panel of ten DNA methylation biomarkers, by assessing candidate gene markers from formalin-fixed paraffin-embedded (FFPE) tissues. With the aim of using the assay in low resource countries, they piloted an automated system for quantifying DNA methylation from cells obtained from fine needle aspiration. The team were able to achieve diagnostic results within 5 hours with a sensitivity of 96% and a specificity of 90% to distinguish tumor from benign tissue. This important study showcases the potential of DNA methylation-based biomarkers to provide rapid and accurate information about a patient’s cancer, to enable equitable clinical implementation.

#### 4.1.2. Biomarkers for disease sub-typing

Current management strategies for breast cancer are informed by a patient’s subtype [64]. Early studies established an association between methylation and hormone status [90–92]. Further studies have corroborated the findings of distinct methylation profiles based on breast cancer subtypes [93–96]. Moreover, through the first application of quantitative trait loci analysis between DNA methylation and gene expression, Fleischer et al. [97] identified that DNA methylation at enhancer regions and at *ER*α, *FOXA1* and *GATA3* binding regions is breast cancer subtype-specific. These studies provide evidence that DNA methylation signatures could be identified for different breast cancer subtypes.

Due to the relative rarity of TNBC, little methylation biomarker work has been conducted in the context of defining TNBC. Branham and colleagues [98] analyzed the methylation status of 110 CpG probes located across 69 cancer-involved genes, to identify a 16 gene methylation signature that was specific to TNBC compared to non-TNBC samples. Further exploring the TNBC epigenetic signature, Stirzaker and colleagues [99] utilized HM450K data from The Cancer Genome Atlas (TCGA) to compare the methylome of TNBC samples to ER+ and ER- breast cancers. This study identified 282 TNBC-specific probes, corresponding to 36 TNBC-specific genomic regions (defined by containing at least three HM450K probes). Additional studies have reinforced that TNBC possesses a unique epigenetic signature from non-TNBC tumors [100,101].

Another example where DNA methylation has the potential to distinguish and provide a new understanding of the molecular basis of different breast tumor subtypes is the rare phyllodes tumors, comprising approximately 1% of all breast tumors [102]. Phyllodes tumors present a significant diagnostic challenge due to the limited molecular characterization of the disease and reliance upon histological examination for diagnosis [103]. However, recent work from Meyer and colleagues [104] demonstrated that phyllodes tumors have a distinct methylome profile compared to histopathologically similar tumors, namely sarcomas and metaplastic breast cancers. The study also identified 53 differentially methylated regions (DMRs) capable of distinguishing malignant from nonmalignant phyllodes tumors. These results underscore the potential clinical utility of methylation biomarkers as a more accurate diagnostic biomarker for phyllodes, addressing a critical need in the field [102].

Defining subtype-specific methylation can provide further understanding of the molecular basis of breast cancer subtypes and aid in the development of targeted therapeutics. However, numerous studies have underscored the heterogeneity present even within breast cancer subtypes [72,93]. Importantly, Conway and colleagues [93] identified specific methylation events that were associated with more aggressive tumor phenotypes, independent of pathology defined subtype. This indicates that DNA methylation may offer insights beyond current diagnostic methods, with the potential to provide, prognostic and predictive information, which will be explored in subsequent sections of this review.

### 4.2. Predictive biomarkers

#### 4.2.1. Chemotherapy

Adjuvant chemotherapy, administered following tumor resection, is a mainstay for the treatment of breast cancer and has been unequivocally demonstrated to improve overall and disease-free survival [105]. These same benefits are also observed in the neoadjuvant setting, along with additional benefits, including broader surgical options and potential breast conservation [106]. However, it is important for clinicians to understand which patients will respond to a given neoadjuvant chemotherapy (NACT) regimen due to the high toxicity and adverse events associated with chemotherapy treatments [107].

The use of DNA methylation biomarkers to predict response to chemotherapy in breast cancer has been of interest as early as 2009 when Hartmann and colleagues [108] investigated biomarkers to predict outcome to adjuvant anthracycline-based chemotherapies in ER+, HER2- patients. They identified methylation at 15 CpG island gene promoters that correlated with treatment response. Importantly, this panel of gene promoter methylation provided additional information to established clinical determinates of outcome such as stage and grade. Within this study, the authors highlighted the predictive ability of *PITX2* methylation, that is, *PITX2* CpG island promoter hypermethylation associates with a high risk of distant recurrence and a poor response to chemotherapy. Further research has corroborated the predictive ability of *PITX2* methylation for adjuvant chemotherapy response in ER+, HER2- breast cancer in a retrospective study [109]. These promising results have culminated in the development of a commercialized *in vitro* diagnostic test, the *Therascreen PITX2 RGQ PCR kit*, a quantitative *in vitro* methylation-specific real-time PCR test [110], that predicts the response of ER+, HER2- breast cancer patients to anthracycline-based chemotherapy, reducing the risk of over-treatment [111,112]. However, more aggressive subtypes of breast cancers including HER2+ and TNBC do not benefit from this assay.

In the TNBC setting, patients rely heavily upon chemotherapy treatment [106]. However, approximately 40% of TNBC patients fail to, or only partially respond to, NACT [113]. To date, candidate gene approaches for biomarker discovery have highlighted an association between *ERα* promoter hypermethylation and cisplatin resistance *in vitr*o, through drug-sensitivity testing on primary TNBC cells [114]. In retrospective patient studies, high *PITX2, FERD3L* and *TRIP10* methylation was associated with a good response to anthracycline and/or taxane-based chemotherapy regimens in non-metastatic TNBC patients [115,116]. Notably, the finding that *PITX2* hypermethylation predicts a good response to chemotherapy in TNBC patients [115] is the opposite of the relationship between *PITX2* methylation and chemotherapy response in non-TNBC patients [108,109].

The complexity of developing suitable candidate DNA methylation biomarkers is demonstrated by the example of *BRCA1* CpG island promoter methylation and its differential predictive value according to chemotherapy type. One study by Tutt and colleagues [117] studied carboplatin responses in comparison to standard of care (docetaxel) in TNBC focusing on whether aberrant *BRCA1/2* methylation contributed to treatment outcomes. The authors found no associations between *BRCA1* methylation and response to carboplatin and the authors concluded that patients with advanced TNBC would not benefit from characterization of *BRCA1* methylation to inform treatment with carboplatin. In contrast, Ignatov and colleagues [118] observed that in patients with TNBC receiving anthracycline-based chemotherapy, *BRCA1* methylation was associated with longer disease-free survival and in turn was a valuable predictor of response to therapy. Studies have continued to investigate and contend the utility of *BRCA1* methylation status as a predictive biomarker [119–121].

Beyond candidate gene studies, Meyer et al. [122] took a genome-wide approach to profile DNA methylation of TNBC biopsy samples collected at diagnosis, mid-chemotherapy and total-excision post-chemotherapy, from patients receiving NACT of FEC100 (fluorouracil, epirubicin and cyclophosphamide) and docetaxel. The authors identified nine ‘response-DMRs’ at diagnosis that were associated with a lack of response to NACT and, interestingly, there was no observed change in methylation at these regions following treatment in both the responders and non-responders. Sigin and colleagues [123], undertook a similar genome-wide sequencing approach for TNBC patients receiving NACT with doxorubicin, cisplatin and paclitaxel and identified 290 differentially methylated genes differentiating responders and non-responders. Selecting markers enriched for specific biological processes, they identified the top 11 markers with the strongest predictive ability. However, none of the regions overlapped between these two studies, potentially due to the different NACT regimens used. Together, this illustrates the potential value of DNA methylation biomarkers to predict response to NACT for TNBC, although the identification of biomarkers to specific NACT regimens may be warranted.

#### 4.2.2. Endocrine therapy

Approximately 70–80% of breast cancers are ER+ and/or PR+ [124]. In such hormone-sensitive breast cancers, activation of hormone receptors by estrogen and progesterone stimulate cell growth [125]. In turn, hormone/endocrine therapies have been developed to inhibit the growth of tumors and have become a mainstay treatment in managing hormone-sensitive breast cancers [126]. However, up to 40% of patients receiving endocrine therapies will develop treatment resistance [127]; thus, targeted biomarkers are needed to determine patients most likely to respond to endocrine therapy and those patients for whom alternate therapies need to be considered.

A foundational study in this area by Widschwendter et al. [92], demonstrated that methylation of the *ESR1* gene promoter, encoding estrogen receptor α (ER), was a strong predictor of response to the anti-estrogen therapy, tamoxifen. Indeed, *ESR1* methylation outperformed hormone receptor status, determined by IHC, in predicting treatment response. Martens and colleagues [128] strengthened the association between methylation status and response to tamoxifen by identifying a panel of 10 genes where associated CpG island promoter methylation was predictive of treatment response, within which *PSAT1* methylation was the strongest predictor of tamoxifen sensitivity. As with chemotherapy regimens, *PITX2* methylation has also been identified as a predictor for endocrine therapy response [129,130]. Other candidate gene studies have identified promoter regions where methylation status is associated with tamoxifen resistance, including *PAX2* [131] and others that have been investigated in *in vivo* models: *SALL2* [132], *GREB1* [133] and *MMP1* [134].

More recent research in this area has focused on ER enhancer-associated methylation, in part due to a study by Stone and colleagues [135] which demonstrated that DNA hypermethylation in ER+ endocrine resistant cell lines predominantly occurs at estrogen-responsive enhancer elements. Methylation levels at 38% of estrogen-responsive enhancer elements negatively correlated with gene expression of the genes with which they were most closely associated, providing a potential novel mechanism by which endocrine response is ablated in ER+ breast cancer, as well as highlighting the potential of *ESR1* methylation at enhancer regulatory elements as a predictive biomarker. Reinforcing this, fulvestrant resistant cell lines have been shown to have dynamic methylation changes at alternative promoter regions of the *ESR1* locus [136]. *ESR1* methylation has been utilized in a circulating tumor DNA (ctDNA) assay, for ER+/HER2- breast cancer patients receiving a combination treatment of everolimus (an mTOR inhibitor) and exemestane (an aromatase inhibitor) [137]. The study found that *ESR1* methylation in circulating tumor cells and ctDNA was associated with a lack of response to the combination treatment, suggesting the potential clinical utility of *ESR1* methylation as a predictive ctDNA biomarker.

#### 4.2.3. Immunotherapy

As a relatively new field, immunotherapies offer significant promise as a novel treatment strategy, whereby the immune system is stimulated to attack cancer cells [138]. However, the current reports suggest that only 5–20% of breast cancer patients respond to monotherapy treatment with immunotherapies [139]. Further, immune-related adverse events are common, with severe adverse events occurring in 20–60% of cancer patients [140]. Together, the lack of response and adverse events associated with immunotherapies highlights the need for effective predictive biomarkers for their use in breast cancer patients.

Immunotherapies are still an emerging treatment for breast cancer; consequently, few studies have investigated DNA methylation biomarkers in this context. Kim and colleagues [141] demonstrated the predictive capacity of baseline DNA methylation at LINE-1 elements in ctDNA from breast cancer patients undergoing nivolumab (programmed cell death protein-1 (PD-1) inhibitor) treatment. This approach was more effective in predicting therapeutic response to nivolumab than current traditional biomarkers such as tumor mutational burden and *PD-L1* expression. In another candidate gene study, Luo et al. [142] observed that hypomethylation of *LRRC3B* (a tumor suppressor gene) in breast tumor tissue was associated with better overall survival in patients receiving an anti-PD1 treatment. While the findings of these studies need to be verified in larger cohorts, they highlight the emerging potential of DNA methylation biomarkers in guiding patient stratification for immunotherapy.

### 4.3. Prognostic biomarkers

While predictive biomarkers can inform whether a treatment is likely to be effective, prognostic biomarkers have a more nuanced, complementary role, informing clinicians as to the likely course of the disease [79]. Prognostic biomarkers exist for a range of outcome measures, including recurrence, progression and overall survival, balanced against the absolute benefits of the treatment [143]. Existing molecular and pathological biomarkers/approaches have been deeply beneficial for informing a patient’s prognosis, however there is need for improvement. This is particularly evident when considering that the most widely used gene expression profiling tests, the 21-gene Oncotype DX and the 70-gene MammaPrint, do not have prognostic utility in patients with TNBC [144,145].

#### 4.3.1. Genome-wide approaches

Alterations in the DNA methylation landscape associated with disease prognosis have been widely researched in hormone receptor-sensitive breast cancers. de Ruijter and colleagues [146] comprehensively summarized findings from 72 publications investigating prognostic DNA methylation biomarkers. They identified that CpG island promoter methylation of *RASSF1, BRCA1, PITX2, CDH1, RARB, PCDH10* and *PGR*, as well as a panel of hypermethylated genes consisting of *GSTP1, RASSF1* and *RARB*, had been associated with poor clinical outcomes in more than one study. Of these, *RASSF1* is the most frequently noted methylation marker [146] and its isoform *RASSF1A* is a known tumor suppressor protein that becomes inactivated in multiple cancers [147]. However, whilst the intersection of biomarkers found in multiple studies is promising, there are many markers that have not been validated in an independent study [146].

One reason for the inconsistency in study results may be differences in study design, such as the selection of a study endpoint and clinical follow-up time. For example, some studies define endpoint by overall survival whilst others use disease recurrence, which can take multiple forms including locoregional recurrence, distant recurrence or second primary disease [146]. Moreover, some studies concentrate on short-term follow-up, as early as 3 years from diagnosis [148], whilst others take a longer term approach focusing on 10 year follow-up [149]. To strengthen their clinical translatability, it is vital for studies to report the endpoint used to allow for replication across studies which, concerningly, only 85% of the studies investigated by de Ruijter and colleagues included [146]. Disparities across studies could also be due to the subtype of breast cancer investigated in the study, as the common approach for many studies is to identify biomarkers for one subtype [76,150,151]. Thus, while prognostic biomarkers are emerging for breast cancer, they need to be specific to endpoint and subtype and require validation in relevant cohorts/datasets.

Recently, Zarean et al [152] sought to validate previously reported CpG sites associated with survival in breast cancer, from five studies that used TCGA data in their CpG discovery. The authors identified partial replication of individual CpGs (9 out of 22), including *HOXD9*, *C17orf93*, *TDRD10*, *AHCYL2*, *SH3PXD2A*, *KCNS3*, *MZF1*, *ELAC1*, *TSPAN15*, of which the strongest association with survival was observed for *C17orf93*. In addition, the authors sought to replicate multi-CpG signatures from 3 studies. They identified replication of 2 out of the 3 signatures, which comprised 16 CpGs and 28 CpGs in their panels. This study represents an important effort in the necessary replication of candidate DNA-methylation-based biomarkers and reinforces the need for detailed reporting of study methods.

TNBC has the greater risk of disease recurrence and reduced overall survival compared to non-TNBC patients [153] meaning that accurate prognostication could have a greater impact on patient survival in this patient population than in other breast cancer subtypes. The need for TNBC-specific prognostic biomarkers is compounded by the fact that current staging does not stratify TNBC well, largely due its propensity for hematogenous rather than lymphatic spread [154]. Stirzaker and colleagues [99] were the first to demonstrate the prognostic potential of DNA methylation in TNBC. Through unsupervised clustering of 4,987 HM450K probes that were differentially methylated between TNBC and normal samples, the authors defined three distinct clusters of TNBC patients with low, medium and high methylation. Individuals within the lowest methylated group had better prognosis, defined by overall survival, compared to the medium and highly methylated clusters. Moreover, the study identified 17 differentially methylated regions associated with survival: 14 at which increased methylation was associated with poor prognosis and 3 at which increased methylation was associated with good prognosis [99]. In another study, focusing on distant disease recurrence as an endpoint, Fackler and colleagues [155] identified that higher levels of methylation in 100 candidate CpG sites was associated with a greater likelihood of 5-year recurrence in TNBC. Sub-setting the 100 marker panel, a smaller panel of 30 probes was also effective at distinguishing recurrent from non-recurrent samples. Importantly, this association was independent of whether the patient received treatment with chemotherapy. Other genome-wide studies have investigated the association between overall survival and methylation; however, presently there is no overlap between identified loci or genes across studies [156,157].

#### 4.3.2. Tumour microenvironment-based approaches

It is now widely recognized that solid tumors exist within a tumor microenvironment (TME) that is comprised of diverse cell types that can contribute to disease progression [158]. TNBC and HER2+ breast cancer subtypes harbor a more abundant tumor infiltrating lymphocyte (TIL) population compared to hormone receptor-sensitive breast cancers [158] and it is now understood that the presence of TILs is a positive prognostic biomarker in these subtypes, particularly for early-stage TNBC [159–161].

In healthy cells, DNA methylation patterns are highly cell type-specific [162]. Therefore, DNA methylation profiling of a sample can be used to estimate cellular proportions [163]. This raises the question of whether DNA methylation profiling can be used to provide a more objective and accurate measurement of TIL proportions in breast cancer subtypes, and therefore be used as a prognostic biomarker. Indeed, several studies have developed methods to deconvolute immune cell composition within tumor and blood samples based on their methylation signature [163,164]. Extending this, Jeschke and colleagues [165] applied a random forest machine learning algorithm to define a TIL specific methylation signature (MeTIL). The study identified 5 CpGs located in the promoter regions of *PTPRCAP, INA, SEMA3B, KLHL6* and *RASSF1* at which methylation was associated with TIL presence, predominantly measuring T cells, B cells and NK cells. Moreover, they found that the MeTIL signature held prognostic value in a cohort of TNBCs; however, when applying the MeTIL signature to pan breast cancers from TCGA, no differences in survival were observed [165], potentially explained by the heterogeneity of the TCGA cohort.

Other studies have utilized a different approach whereby DNA methylation signatures associated with prognosis have been correlated with TME estimates from histopathological assessments. One such study by Zhang and colleagues [166] developed an ‘epigenetic risk score’ based on the methylation status of 10 prognostic, breast cancer-specific CpG sites from TCGA samples. The authors proposed that their signature could indicate an inflamed TME, because the risk score was negatively correlated with histopathological measures of tumor-associated infiltrating immune cells, T cell inflamed score and other TME estimates. Li and colleagues [167] determined that DNA methylation of *neurofilament medium* (*NEFM*) loci was associated with poor prognosis, and indeed, *NEFM* DNA methylation was negatively correlated with immune infiltration. Similarly, *IDO1* promoter hypomethylation in the basal subtype has been associated with a favorable prognosis and immune cell infiltration [168]. Whilst these studies demonstrate an exciting connection between methylation measurements and the TME, the results were derived from methylation data alone and the link between their candidate CpG sites and their biological mechanisms in relation to immune cells, requires further investigation.

### 4.4. Circulating tumour DNA methylation biomarkers

**Figure 4.**
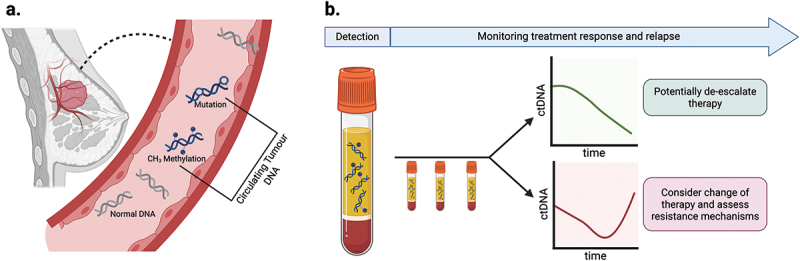
Circulating tumor DNA (ctDNA) as liquid biopsy for breast cancer.

Cancer-associated methylation patterns can also be detected in ctDNA, which describes DNA fragments actively secreted and/or originating from necrotic or apoptotic cancer cells [169] (Figure 4(a)). ctDNA retains the genomic profile of primary tumors, including its complement of epigenetic modifications, mutations and copy number variations [170]. Indeed, with its half-life of 16 minutes to 2.5 hours, ctDNA is a source of real-time tumor information [169]. ctDNA can be found in a range of biological samples, including blood, urine, cerebrospinal fluid and pleural fluid [171–173]. Tests on these samples, also known as ‘liquid biopsies,’ offer a less invasive and potentially cost-effective alternative to traditional tumor biopsies [174].

(a) ctDNA from necrotic and apoptotic cancer cells capture the epi/genomic profile of the primary tumor, allowing DNA methylation changes and mutations associated with the tumor to be detected in the blood. (b) In the plasma isolated from blood samples, tumor-specific epigenetic marks can allow for the quantitative detection of ctDNA. Serial measurements of ctDNA levels throughout the patient’s treatment, can enable real-time monitoring of the patient’s tumor burden and inform their treatment plan. Figure created in BioRender. Williams, S. (2025) https://BioRender.com/0cg2ll5

A substantial number of studies have demonstrated the feasibility of detection and sequential enumeration of ctDNA presence in breast cancer [81,175–177]. Incorporating DNA methylation signatures within ctDNA assays could further improve early detection and enable more precise monitoring of the tumor [170,178] (Figure 4(b)). Indeed, the potential of methylation-based ctDNA tests has been demonstrated by the development of the Galleri^TM^ test, currently available as a multi-cancer early detection test, capable of detecting the presence of the disease and the tumor’s tissue of origin [171,179–181] (Table 2).

**Table 2.** Summary of biomarker tests that are commercially available or implemented in prospective trials.

Biomarker test name	Key studies	Methylation target	Clinical use	Breast cancer subtype	Sample	Detection Method	Development Stage
*Therascreen PITX2 RGQ PCR* kit	Napieralski et al [110] Schricker et al [112]	Three CpG sites of the *PITX2* gene promoter 2	To aid clinicians in the prediction of response to adjuvant anthracycline-based chemotherapy, with or without endocrine therapy.	ER+, HER2-, LN+	FFPE Tumour tissue	qMSP	The kit is commercially available. Whilst it is CE-certified for use in Europe it is not FDA approved.
Galleri, GRAIL test	Liu et al [171] Klein et al [179] Schrag et al [180] Nicholson et al [181]	1 116 720 CpGs covering 103 456 genomic regions	Diagnostic test for early detection of multiple cancers ( > 50 cancers).	All breast cancer subtypes	Plasma	TBS	The Galleri, GRAIL test has been utilized in two currently published prospective trials. There are ongoing prospective trials utilizing the Galleri,GRAIL test: (ClinicalTrials.gov, NCT05155605, NCT05673018) and (ISRCTN, ISRCTN91431511) The Galleri test is currently commercially available however it is not FDA approved.
mDETECT	Cristall et al [182]	53 CpG sites covering 47 genomic regions	To detect and monitor patients with metastatic TNBC.	TNBC	Plasma	qMSP	Currently, the only published results are from a retrospective study, however there is an ongoing trial for the mDETECT assay: (ClinicalTrials.gov, NCT05804578)
LBx-BCM	Visvanathan et al [183] Fackler et al [184]	CpG sites in *AKR1B1, TM6SF1, ZNF671, TMEFF2, COL6A2, HIST1H3C, RASGRF2, HOXB4, RASSF1*	To detect disease progression following treatment initiation in patients with metastatic breast cancer.	All Subtypes	Plasma	qMSP	Implemented in a prospective study design using samples from the prospective trial, TBCRC 005. This cartridge-based system is referred to as the LBx-BCM.

PCR: Polymerase Chain Reaction, qMSP: real-time DNA methylation specific PCR, TBS: Targeted Bisulfite Sequencing, ER: Estrogen Receptor, HER2: Human Epidermal Growth Factor Receptor 2, LN: Lymph Node, TNBC: Triple Negative Breast Cancer, FDA: US Food and Drug Administration, FFPE: Formalin Fixed Paraffin Embedded.

Early detection in the relapse setting and monitoring of breast cancer is key to improving the outcomes of patients [64]. In metastatic TNBC, Cristall and colleagues [182] developed the methylation DETEction of Circulating Tumour DNA (mDETECT) assay to analyze plasma samples, incorporating 53 probes covering 47 regions. mDETECT was able detect a tumor with a sensitivity of 76% and a specificity of 100%, moreover, the assay offered better detection than CA 15–3 (the most widely used serum tumor marker in breast cancer [185]). In non-metastatic TNBC tissue samples, Manoochehri and colleagues utilized a machine learning approach to identify 6 differentially methylated regions (*SPAG6, IFFO1, SPHK2, CPXM1, LINC01606, TBCD/ZNF750*) that could discriminate TNBC patients from healthy controls [186]. To validate these DMRs in plasma, the group quantified the un-/methylated copies of these DMRs in a droplet-digital PCR (ddPCR) assay and confirmed that their panel was capable of discriminating TNBC samples from controls.

A comprehensive body of work has culminated in the development of the cMethDNA assay for the detection and monitoring of metastatic breast cancer across subtypes [183,187,188]. Initially, Fackler et al. [187] developed a 10 gene promoter panel (*AKR1B1, COL6A2, GPX7, HIST1H3C, HOXB4, RASGRF2, TM6SF1, ARHGEF7, TMEFF2* and *RASSF1*) whose methylation status was capable of detecting cancer in the serum of patients. Moreover, the assay was reflective of the patient's response to chemotherapy [187]. In a larger prospective study, six of the ten genes in the original cMethDNA panel were a strong predictor of survival outcomes in metastatic breast cancer [188]. Recently, nine of the ten genes within the cMethDNA panel have been implemented in an automated DNA methylation cartridge assay, (Liquid Biopsy for Breast Cancer Methylation, LBx-BCM) for the detection of metastatic breast cancer, achieving high sensitivity (83%) and specificity (92%) [184]. To analyze the clinical utility of the LBx-BCM assay, Visvanathan et al. [183] assessed methylation in plasma collected at baseline, week 4 and week 8 post-treatment initiation. Strikingly, the detection of high cumulative methylation levels as early as week 4 was associated with worse overall survival as well as progressive disease. Collectively, these studies show the promise of the LBx-BCM assay as a clinical tool for monitoring metastatic breast cancer.

In contrast to detecting tumor-specific methylation patterns, Moss and colleagues [189] identified genomic loci of normal breast epithelium with the rationale that elevated tissue-specific loci in cell-free DNA could indicate the presence of breast cancer. The authors identified three regions (*KRT19, LMX1b*, and *ZNF296*) with unique methylation in breast epithelial cells, capable of identifying metastatic breast cancer from plasma samples and reflective of patient response to chemotherapy. Another unique approach to the detection of breast cancer has included assessment of genome-wide hypomethylation patterns in plasma samples [190]. Although attractive for diagnostic implementation given its low sequence depth, this approach has not seen clinical implementation.

Despite the potential of DNA methylation markers in ctDNA, there are significant challenges affecting the implementation of these biomarkers, and liquid biopsies more generally, into routine clinical practice [191]. One major challenge for the development of ctDNA assays is that the concentration of ctDNA is relatively low in early-stage disease. This is exemplified in a clinical validation study for the Galleri test that identified that whilst the overall sensitivity for breast cancer was 30.5%, stage 1 disease was detected with a sensitivity of 2.6% compared to 90.9% for stage 4 disease [179]. In addition, liquid biopsies from blood are inherently mixed, containing cfDNA as well as other analytes such as leukocytes, which may confound ctDNA signals [173]. Specific isolation techniques such as size selection or exclusion of CD45 positive leukocytes are critical [192]. Alternatively, given the cell-type specifity of DNA methylation, tumor methylation profiles can be compared against normal blood leukocyte methylation profiles to identify tumor-specific gene regions in ctDNA assay design, a method already successfully adopted for ctDNA detection [193–195].

## 5. Conclusion

This review highlights the significant progress achieved over recent decades in the development of DNA methylation-based biomarkers for breast cancer care. Whilst many studies to date are retrospective, some biomarkers highlighted in this review have achieved further advancement to commercialization or implementation in prospective clinical trial (as summarized in Table 2). The integration of advanced research methodologies and bioinformatic tools, alongside their incorporation into liquid biopsy platforms and ctDNA assays, offers promising opportunities for these biomarkers to be more widely adopted in clinical practice. Furthermore, the expanding knowledge of the TME presents new avenues for the discovery of robust DNA methylation biomarkers. Further steps are required to fully realize the potential of DNA methylation-based biomarkers. Addressing the challenge of replicability is essential, which necessitates the comprehensive reporting of study methodologies and findings. Moreover, accounting for population and study heterogeneity is critical to improve the generalizability and equitable use of these biomarkers. With these issues addressed, the integration of methylation-based biomarkers in clinical management offers a promising avenue to improve breast cancer outcomes and quality of life.

## 6. Future perspective

The growing innovation and competition in the field of DNA methylation biomarker research means that we are likely to see improved translation of DNA methylation biomarkers to the clinic, both for breast cancer and other cancer types. Indeed, key barriers to implementation, such as the lack of standardized methodologies and the need for cost-effective assays like ddPCR, are beginning to be addressed. One particularly exciting area of the biomarker clinical research field is in the further development of blood-based biomarkers. This modality offers a unique ability to serially monitor real-time tumor response, of interest to both researchers and clinicians. Importantly, there has also been considerable consumer advocacy for the development of noninvasive tests such as liquid biopsy, motivating the field to act on this need.

## Article highlights

- Aberrant DNA methylation is a hallmark of cancer and can take the form of global hypomethylation in parallel with promoter CpG island hypermethylation. These modifications occur early and are highly pervasive making them suitable for the development of biomarkers that can be utilized across a patient’s cancer journey.
- After decades of research, DNA methylated-based biomarkers are beginning to reach clinical translation. A notable example is the *Therascreen PITX2 RGQ PCR* kit, a DNA methylation-based test, used to predict chemotherapy response in ER+, HER2- breast cancer patients.
- With a growing understanding of the role of the diverse cell types in the tumor microenvironment, DNA methylation biomarkers panels are expanding to account for cell composition. This is proving particularly important in the development of prognostic biomarkers.
- The analysis of blood for ctDNA is opening new avenues for cancer diagnostics. Given the lack of current tools to serially monitor breast cancer patients for relapse, this represents a major stride in the field of breast cancer management.
